# ALASKA NATIVE DEMENTIA CAREGIVERS AND THE INCREASING NEED FOR CULTURALLY SAFE RESOURCES

**DOI:** 10.1093/geroni/igad104.0813

**Published:** 2023-12-21

**Authors:** Jordan Lewis, Steffi Kim, Zayla Asquith-Heinz, Lena Thompson

**Affiliations:** University of Minnesota Medical School, Duluth campus, Duluth, Minnesota, United States; University of Alaska Anchorage, Anchorage, Alaska, United States; University of Minnesota Duluth, Duluth, Minnesota, United States; University of Minnesota, Duluth, Minnesota, United States

## Abstract

With the rate of dementia increasing among Alaska Native people and the tribal health system ill-equipped to meet the challenges associated with dementia care, there is an urgent need to better understand cultural perspectives on dementia caregiving and what states and communities can do to support their caregivers. Despite the fact caring for others is a value and cultural practice for Indigenous communities, and caregivers tend to report fewer challenges and barriers related to care, they still experience barriers and challenges. Guided by Kleinman’s explanatory model of illness, a qualitative, the aim of this study was to gain insight on Alaska Native caregiver experiences in caring for someone living with dementia in communities across Alaska. 21 interviews with Alaska Native dementia caregivers in rural and urban communities were completed. Despite being innovative and resourceful in providing care, caregivers reported the high cost of care, inadequate training of health care providers, and the paucity of culturally safe caregiver training, education, and resources are especially concerning and need to be addressed in a timely fashion. Caregivers in this study recommended the development of basic educational materials to be aware of dementia, its stages, and the available supports, as well as caregiver support and respite services. The recommendations from this study focus on outreach and education for caregivers to improve their access to education, training, and support to reduce isolation, increase their dementia knowledge, expand their awareness of ancillary resources, and inform help-seeking decisions.

